# The Expression of Programmed Death Ligand 1 and Vimentin in Resected Non-Metastatic Non-Small-Cell Lung Cancer: Interplay and Prognostic Effects

**DOI:** 10.3389/fcell.2021.772216

**Published:** 2021-11-30

**Authors:** Sara Bravaccini, Giuseppe Bronte, Elisabetta Petracci, Maurizio Puccetti, Manolo D’Arcangelo, Sara Ravaioli, Maria Maddalena Tumedei, Roberta Maltoni, Angelo Delmonte, Federico Cappuzzo, Lucio Crinò

**Affiliations:** ^1^ Biosciences Laboratory, IRCCS Istituto Romagnolo per lo Studio dei Tumori (IRST) “Dino Amadori”, Meldola, Italy; ^2^ Department of Medical Oncology, IRCCS Istituto Romagnolo per lo Studio dei Tumori (IRST) “Dino Amadori”, Meldola, Italy; ^3^ Unit of Biostatistics and Clinical Trials, IRCCS Istituto Romagnolo per lo Studio dei Tumori (IRST) “Dino Amadori”, Meldola, Italy; ^4^ Azienda Unità Sanitaria Locale (AUSL) Imola, Imola, Italy; ^5^ AUSL Romagna, Ospedale Santa Maria delle Croci, Ravenna, Italy; ^6^ Istituto Nazionale Tumori “Regina Elena”, Rome, Italy

**Keywords:** NSCLC, PD-L1, vimentin, immune infiltrate, non-metastatic (M0) patients

## Abstract

Programmed death ligand 1 (PD-L1) is an immune checkpoint with a role in cancer-related immune evasion. It is a target for cancer immunotherapy and its expression is detected for the use of some immune checkpoint inhibitors in advanced non-small cell lung cancer patients (NSCLC). Vimentin is a key component of the epithelial-to-mesenchymal transition phenotype. Its expression has negative prognostic effects in NSCLC. In this study, we retrospectively evaluated PD-L1 and vimentin expression in tumor cells, immune infiltrate and PD-L1 positive immune infiltrate via immunohistochemistry in tissue samples from resected non-metastatic NSCLC patients. We explored the interplay between PD-L1 and vimentin expression through Spearman’s correlation test. We performed univariate analysis through the Cox models for demographic and clinico-pathological variables, and also for dichotomized biomarkers, i.e., PD-L1 and vimentin in tumor cells, both with 1 and 50% cutoffs. We used Kaplan-Meier method to estimate the overall survival, comparing both vimentin and PD-L1 positive patients with all the others. We found a weak positive correlation between PD-L1 and vimentin expressions in tumor cells (*r* = 0.25; *p* = 0.001). We also observed a statistically not significant trend towards a shorter overall survival in patients with both PD-L1 and vimentin expression >1% (HR = 1.36; 95% CI: 0.96–1.93, *p* = 0.087). In conclusion, these findings suggest that interplay between PD-L1 and vimentin may exist in non-metastatic NSCLC patients and the positivity of both markers in tumor tissue is associated with a trend towards a worse prognosis.

## Introduction

Lung cancer is still the leading cause of mortality by cancer, despite recent therapeutic advances. In non-small cell lung cancer (NSCLC) 5-year survival rates are limited to 60% in patients with stage II and 36% in those with stage IIIA according to the 8th edition staging by the International Association for the Study of Lung Cancer (IASLC) (Goldstraw et al., 2016). The surgery remains the gold standard treatment for patients in early stages (I-II); it can be considered in selected cases with stage IIIA disease. Post-surgical adjuvant platinum-based chemotherapy is used to achieve a modest improvement of around 5% in 5-year overall survival (OS) in patients who underwent complete resection for stage II and IIIA (Pignon et al., 2008). It can be considered in those patients who underwent resection for stage IB NSCLC if the primary tumor is greater than 4 cm. For unresectable stage IIIA or IIIB disease the treatment of choice is represented by concurrent chemoradiation therapy or, as an alternative, sequential chemotherapy followed by definitive radiotherapy (Postmus et al., 2017). In both cases, concurrent or sequential therapy, a consolidation with durvalumab, an immune checkpoint inhibitor (ICI), is approved according to the results of PACIFIC trial (Antonia et al., 2017). ICIs are currently being evaluated as neoadjuvant/adjuvant therapy in combination with standard treatments (Mielgo-Rubio et al., 2020).

The interaction between Programmed Death-1 (PD–1) and its ligand, Programmed Death–Ligand 1 (PD–L1), is an immune checkpoint with a relevant role in the regulation of anti-tumor immune response. These molecules are expressed in tumor-infiltrating immune cells and tumor cells, respectively. In some cases, the expression of PD-L1 evaluated through immunohistochemistry (IHC) helped to provide a predictive biomarker for ICI. Specifically, it allowed patients to be selected for upfront pembrolizumab, an anti-PD-1 ICI, among those with metastatic NSCLC which expressed PD-L1 > 50%, before the approval of chemoimmunotherapy as first-line treatment.

Epithelial–mesenchymal transition (EMT) may be associated with higher aggressiveness in NSCLC.

It is characterized by the reduction of epithelial features and an increase of mesenchymal ones. In this biological condition tumor cells are more invasive and maintain longer survival leading to higher numbers of circulating tumor cells and metastases (Francart et al., 2018). EMT characteristics include a higher expression of vimentin EMT-related transcription factors, and a remodeling of cell–cell contacts (Tsoukalas et al., 2017). The EMT phenotype was related to PD-L1 upregulation by the combined effect of TGF-β1 and TNF-α. This could be due to the action of TNF-α on NF-κB stimulation, which increases EMT induction by TGF-β1. NF-κB inhibition also blocks PD-L1 expression (Asgarova et al., 2018). These results are supported by the fact that TGF-β1 and TNF-α induce global DNA demethylation, including the demethylation of the PD-L1 promoter, which causes higher PD-L1 expression.

Moreover, vimentin has been described as a negative prognostic marker in various cancers (Dongre and Weinberg, 2019), and also in NSCLC, as extensively documented by numerous studies, then pooled in a meta-analysis (Ye et al., 2016).

In this paper we evaluated the interplay between PD-L1 and vimentin in a wide population of non-metastatic NSCLC patients. We also explored a potential role of immune infiltrate in tumor tissue and analyzed the prognostic impact of these combined markers.

## Materials and Methods

### Study Design

To investigate the association between Vimentin and PD-L1 expression and immune infiltrate and their separate and combined effects in terms of OS, retrospectively retrieved data for patients consecutively treated at Area Vasta Romagna (AVR) since July, 1997, were used. Our hypothesis is that the increase of vimentin expression levels is associated with an increase of PD-L1 expression levels. We also hypothesized that the patients with both Vimentin and PD-L1 positive IHC expression had a worse prognosis in terms of OS. Second aim of the study was to explore the role of immune infiltrate in terms of quantity and percentage of PD-L1 positive immune cells on OS.

### Patients

Eligibility criteria were ≥18 years old, histological diagnosis of non-metastatic NSCLC (Stage I-IIIA defined by using the latest version of staging system). At least one primary tumor specimen had to be available. From the clinical records we extracted information about histology, stage at diagnosis, date of diagnosis, type of radical surgery, adjuvant chemotherapy, adjuvant radiotherapy, date of death or date of last follow-up visit.

Given that the setting of patients analyzed is early stage NSCLC, the assessment of genetic testing was not required.

The Ethics Committee of AVR reviewed and approved the study protocol (NCT03078959). Patients provided written informed consent according to Italian privacy law and following the principles laid down in the Declaration of Helsinki.

### Immunohistochemistry

NSCLC obtained during surgery was fixed in neutral buffered formalin and embedded in paraffin. Four-micron sections were mounted on positive-charged slides (Bio Optica, Milan, Italy). Biomarker evaluations were done according to European Quality Assurance guidelines. PD-L1 and Vimentin Immunostaining expression was performed using PD-L1 SP263 and Confirm anti-Vimentin V9 (Ventana Medical Systems) antibody clones by Ventana BenchmarkXT staining system (Ventana Medical Systems, Tucson, AZ, United States) with the Optiview DAB Detection Kit (Ventana Medical Systems).

For their detection, tissue sections were incubated for 16 min with prediluted antibodies by the supplier. Sections were incubated for 16 min and automatically counterstained with hematoxylin II (Ventana Medical Systems). Placenta was used as positive control for both the biomarkers. Membranous biomarker positivity was detected and semiquantitatively quantified as the percentage ratio between immunopositive tumor cells and the total number of tumor cells.

We defined immune infiltrate considering mainly the tumor infiltrating lymphocytes (TILs). PD-L1 positivity was detected both on tumor cells and on the immune infiltrate. We considered the amount (%) of the whole immune infiltrate on the total of the cells present in the sample. The PD-L1 positivity on immune infiltrate was evaluated in terms of percentage of PD-L1 positive immune cells on the total number of immune cells without subtyping the TILs identified by using only morphological criteria, due to the low number of sections available to perform further IHC detections.

Vimentin expression has been evaluated only on tumor cells given that its expression in the cells of interstitial area is known to be ubiquitous.

All samples were evaluated by 2 independent observers and any disagreement (>10% of positive cells for the different markers) was resolved by consensus after joint review using a multihead microscope.

### Statistical Analysis

Data were summarized using mean ± standard deviation (sd), median and minimum and maximum values, for continuous variables. Categorical variables were reported as natural frequency and percentage. Correlation among variables was measured through the Spearman correlation coefficient.

Given the heavily asymmetric distribution of the considered biomarkers, characterized by a “spike at zero,” the association between demographic and clinical covariates as well as with the OS was assessed using dichotomized variables on the 1% value. Patients with expression values lower than 1% were considered “negative” for the biomarkers whereas those with values equal or greater than 1%, were considered as “positive.” Additional exploratory analyses considering a 50% cutoff (“negative” less than 50%, “positive” if equal or greater than 50%) as well as considering the biomarkers as continuous variables were also performed. These cutoffs were chosen because these are commonly used in clinical practice for PD-L1 characterization for the use of pembrolizumab in first- or second-line treatment for advanced NSCLC. The Chi-square test or the Fisher Exact test was used for the association between the biomarker as dichotomous variable and categorical covariates whereas the Wilcoxon-Mann-Whitney or the Kruskal Wallis test was used for the association between the biomarker as continuous variable and categorical covariates.

OS was defined as the time from surgery until death for any cause or last patient visit by April, 2016. The Kaplan-Meier method was used to estimate the OS function whereas the Log-rank test was used for survival curves comparison. Hazard ratios (HRs) and corresponsing 95% confidence intervals (CIs) were obtained applying the Cox proportional hazards model. To investigate the association between the biomarkers as continuous variables, the method proposed by Royston and Lorenz was also applied (Royston et al., 2010; Lorenz et al., 2017; Lorenz et al., 2019). The proportional hazards assumption was tested using Schoenfeld residuals. For all the analyses a two-sided *p*-value <0.05 was considered statistically significant. The analyses were carried with STATA 15.0 (College Station, Texas, United States).

## Results

In this retrospective study we included 247 patients who met the eligibility criteria. The majority of patients were male (79%), former or current smokers (85%), with a non-squamous histology (60%). The mean age at surgery was 68.20 ± 7.85 years. The stages were variably distributed: 36% stage I, 37% stage II, 27% stage IIIA. As regards surgery, the majority of patients (62%) underwent lobectomy. As regards cancer treatments, 17% received neoadjuvant chemotherapy, 10% received adjuvant chemotherapy and none of the patients was treated with post-surgical radiation therapy (Table 1). The mean values of PD-L1 expression in tumor cells, vimentin expression in tumor cells, immune infiltrate, and PD-L1-positive immune infiltrate with standard deviations are reported in Table 2, together with medians and minimum and maximum values. PD-L1 median percentage in tumor cells was equal to zero as well as the vimentin median percentage in tumor cells. The median values for the immune infiltrate and the PD-L1-positive immune infiltrate were equal to 10 and 2, respectively.

**TABLE 1:** Patients characteristics (*n* = 247).

	*N*	%
Gender		
female	51	20.65
male	196	79.35
*missing*	−	
Age at surgery, yrs		
mean ± sd	68.20 ± 7.85	
Median (min-max)	69 (36–87)	
*missing*	−	
Smoking status		
Non-smoker	6	15.38
Former smoker	20	51.28
Current smoker	13	33.33
*missing*	*208*	
Histology		
Non squamous	148	59.92
Squamous	94	38.06
Mixed	5	2.02
*missing*	−	
Grading		
G1	10	4.31
G2	87	37.50
G3	135	58.19
*missing*	15	
Disease stage (8th edition)		
IA	22	11.70
IB	45	23.94
IIA	20	10.64
IIB	50	26.60
IIIA	51	27.13
*missing*	59	
Type of surgery		
lobectomy	153	61.94
bilobectomy	10	4.05
pneumonectomy	38	15.38
Atypical resection	44	17.81
Other	2	0.81
*missing*	−	
Neoadjuvant Chemotherapy		
no	20	83.33
yes	4	16.67
*missing*	223	
Adjuvant Chemotherapy		
no	19	90.48
yes	2	9.52
*missing*	226	
Post-surgery Radiotherapy		
no	21	100.00
yes	−	0.00
*missing*	226	
		

sd: standard deviation; min: minimum; max: maximum.

**TABLE 2:** Descriptive statistics of IHC biomarkers.

	*missing*	Mean	sd	Median	min	Max	IQR
PD-L1+ tumor cells	−	20.76	33.24	0	0	100	30
Vim + tumor cells	−	10.96	23.19	0	0	100	5
Immune infiltrate	12	16.01	14.99	10	1	90	15
PD-L1+ immune infiltrate	12	5.53	9.36	2	0	80	10

IHC: immunohistochemistry; PD-L1: programmed death ligand 1; Vim: vimentin; sd: standard deviation; min: minimum; max: maximum; IQR: interquartile range.

**FIGURE 1:**
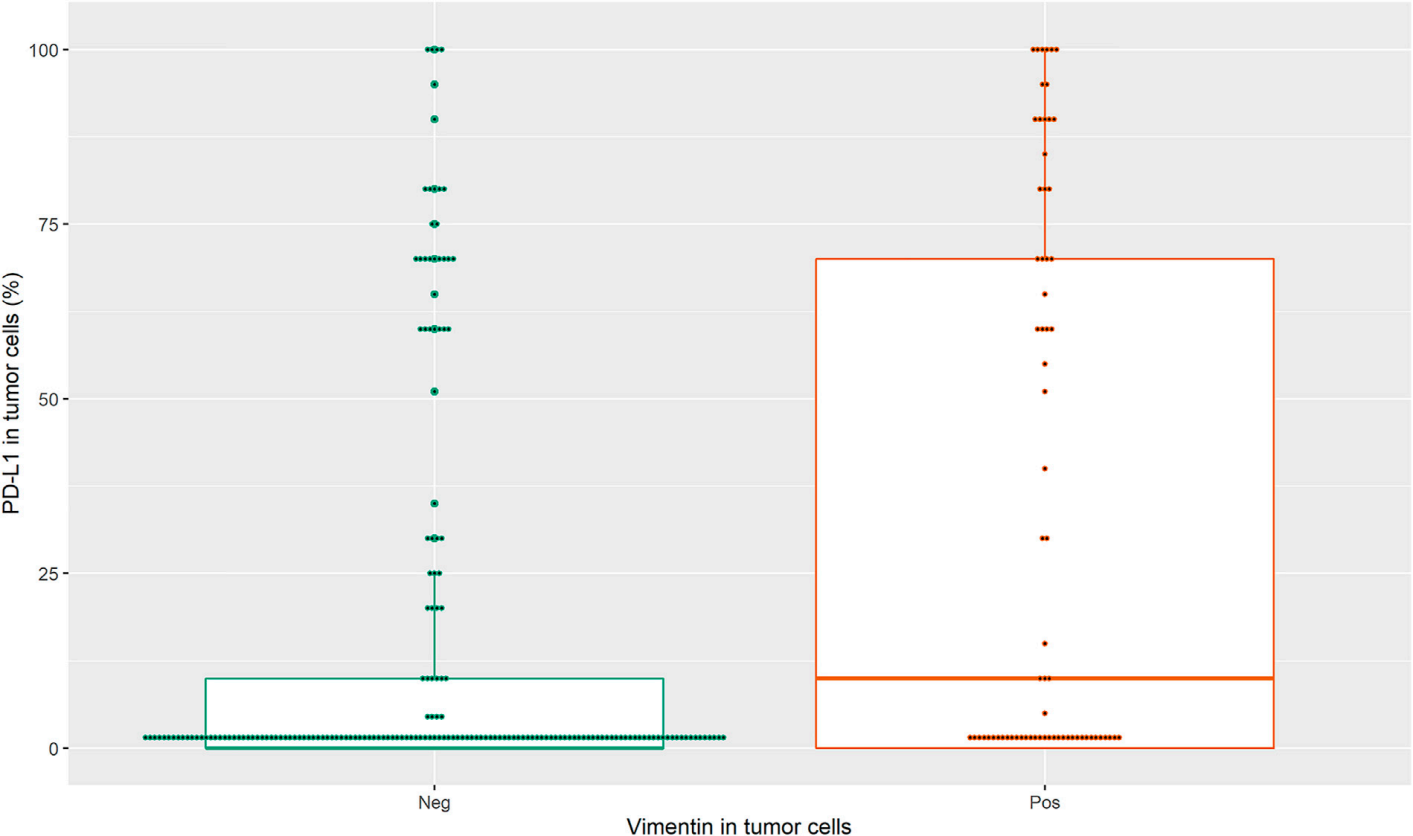
Correlation between the IHC-defined biomarkers i.e. PD-L1 in tumor cells as continuous variable and vimentin in tumor cells as dichotomous variable.

We measured the correlation between the IHC-defined biomarkers as continuous variables through the Spearman correlation coefficient. We found a weak positive correlation between PD-L1 and vimentin expressions in tumor cells (*r* = 0.25; *p* = 0.001). The comparison of PD-L1 expression in tumor cells between high and low vimentin expression (1% of cutoff value) by using Wilcoxon-Mann-Whitney resulted in a statistically significant difference (*p*-value<0.001), Figure 1.

A similar result was obtained using the 50% cutoff for vimentin expression (data not shown).

The tumor cells were negative for PD-L1 or vimentin with both the cutoff values in the majority of patients. Tumor cells were PD-L1-negative in 76% of cases with 50% cutoff and 57% with 1% cutoff. The same cells were vimentin-negative in 89% of cases with 50% cutoff and 72% with 1% cutoff. For the samples from 12 patients the information about immune infiltrate was missing. However, almost all cases (97%) were negative for immune infiltrate with 50% cutoff value, but all the samples had an immune infiltrate ≥1%. In 99% of cases the immune infiltrate had a PD-L1 expression <50%, but with 1% cutoff value PD-L1-positive immune infiltrate was present in the majority of patients (67%) (Table 3).

**TABLE 3:** Distribution of biomarkers’ values with different cutoffs.

	50% cutoff		1% cutoff	
	*N*	%	*n*	%
PD-L1 in tumor cells				
Negative	188	76.11	140	56.68
Positive	59	23.89	107	43.32
*missing*	−		−	
Vimentin in tumor cells				
Negative	220	89.07	207	71.63
Positive	27	10.93	82	28.37
*missing*	−		−	
Immune infiltrate				
Negative	227	96.60	0	0.00
Positive	8	3.40	235	100.00
*missing*	12		12	
PD-L1+ immune infiltrate				
Negative	233	99.15	78	33.19
Positive	2	0.85	157	66.81
*missing*	12		12	

No significant associations were found between the biomarkers and demographic and clinico-pathological covariates (results not shown).

We also performed an univariate analysis for OS through the Cox models as regards the available demographic and clinico-pathological variables, including gender, age, smoking status, histology, grading and disease stage, and type of surgery. We found statistically significant differences for age (<70 vs. ≥ 70 years), grading (G1 vs. G3) and disease stage (I vs. III) (Table 4).

**TABLE 4:** Results from univariate Cox models for demographic and clinico-pathological variables.

	HR (95% CI)	P
Gender		
Female	1 (ref)	
Male	1.22 (0.88–1.70)	0.239
Age		
<70	1 (ref)	
≥70	1.40 (1.15–1.95)	**0.003**
Smoking status		
non smoker	1 (ref)	
ex smoker	0.64 (0.23–1.78)	0.399
Smoker	0.77 (0.26–2.26)	0.632
Histology		
non squamous	1 (ref)	
Squamous	1.03 (0.78–1.35)	0.848
Mixed	0.98 (0.36–2.65)	0.964
Grading		
G1	1 (ref)	
G2	1.71 (0.83–3.55)	0.148
G3	2.24 (1.09–4.59)	**0.027**
Disease stage		
I	1 (ref)	
II	1.40 (0.97–2.01)	0.072
III	2.15 (1.52–3.32)	**<0.001**

HR: hazard ratio; CI: confidence interval; P: *p*-value; ref: reference.

The bold values refer to statistically significant values (*p* < 0.05).

An univariate analysis for OS was also performed for the dichotomized biomarkers (Table 5). Note that for the immune infiltrate only eight patients had a value ≥ 50% whereas for the PD-L1+immune infiltrate only two patients presented with a value ≥50%. However, we did not find statistically significant differences for the analyzed biomarkers (Table 5). No statistically significant associations for all four biomarkers were observed also when considered as continuous variables (results not shown).

**TABLE 5:** Results from univariate Cox models for dichotomized biomarkers.

	50% cutoff		1% cutoff	
	HR (95% CI)	P	HR (95% CI)	P
PD-L1 in tumor cells				
Negative	1 (ref)		1 (ref)	
Positive	0.95 (0.69–1.30)	0.732	1.01 (0.78–1.30)	0.961
Vimentin in tumor cells				
Negative	1 (ref)		1 (ref)	
Positive	1.14 (0.72–1.81)	0.565	1.19 (0.90–1.57)	0.219
Immune infiltrate				
Negative	1 (ref)		1 (ref)	
Positive	0.66 (0.29–1.48)	0.312	−^a^	
PD-L1+ immune infiltrate				
Negative	1 (ref)		1 (ref)	
Positive	1.33 (0.33–5.37)	0.687	1.19 (0.89–1.58)	0.243

HR: hazard ratio; CI: confidence interval; P: *p*-value; ref: reference.

a HRs, and 95% CIs, could not be estimated because no patients presented with a value < 1%.

**FIGURE 2:**
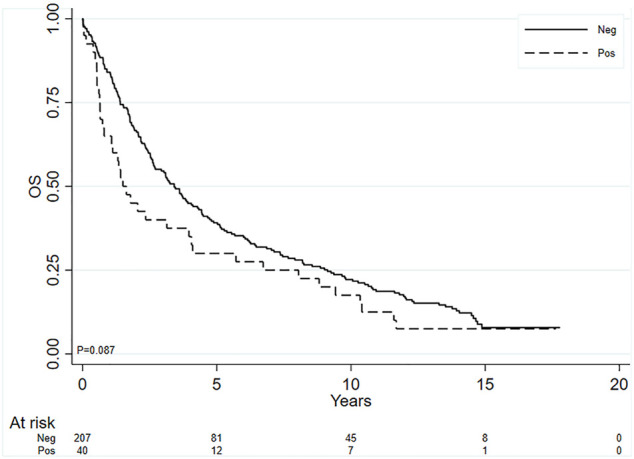
Kaplan-Meier curves for OS, comparing the subgroup of patients with both PD-L1 and vimentin positive in tumor cells (cutoff value ≥ 1% for both the biomarkers) with all the others.

**FIGURE 3:**
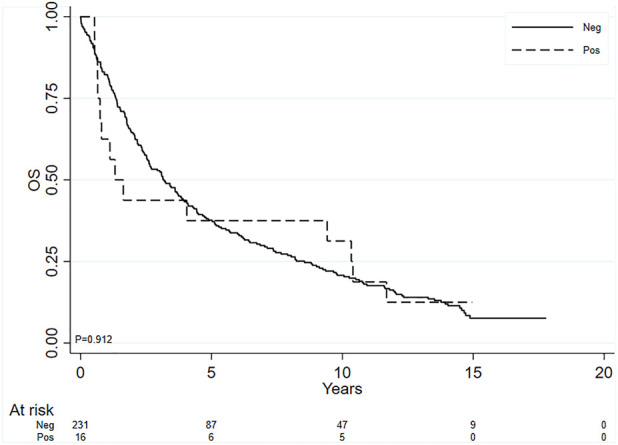
Kaplan-Meier curves for OS, comparing the subgroup of patients with both PD-L1 and vimentin positive in tumor cells (cutoff value ≥ 50% for both the biomarkers) with all the others.

Finally, given that we hypothesized that the patients with both Vimentin and PD-L1 positive (40 patients) could have a worse prognosis, we compared this subgroup of patients with all other patients (*n* = 207). We found a trend even if not statistically significant toward a worse survival in this double positive subgroup using 1% cutoff value for both the biomarkers (HR = 1.36; 95% CI: 0.96–1.93, *p* = 0.087). Figure 2 shows the Kaplan-Meier curves for the two groups. Such a trend in significance disappeared when age and stage were also included in the Cox model. When considering the 50% cutoff no association was observed (HR = 1.03; 95% CI: 0.60–1.77, *p* = 0.912), Figure 3.

**FIGURE 4:**
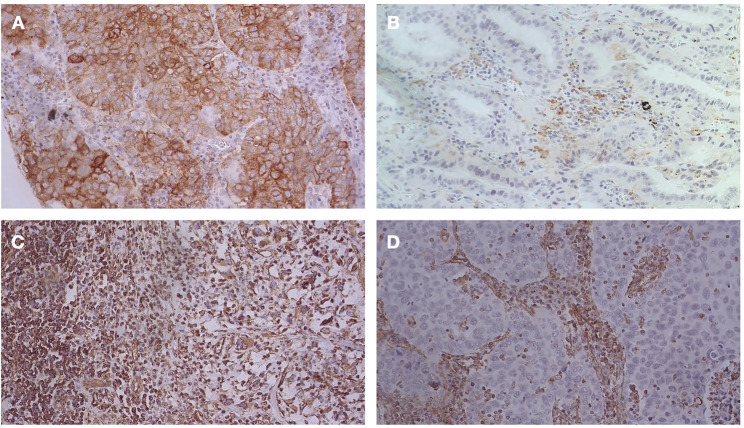
IHC analysis for PD-L1 and vimentin: **(A)** PD-L1 positive in both tumor cells and infiltrate with immune cells (40X magnification); **(B)** PD-L1 positive in infiltrate with immune cells, but negative in tumor cells (40X magnification); **(C)** Vimentin expression observed both in tumor cells and immune cells (20X magnification); **(D)** Vimentin expression observed only in immune cells (20X magnification).

**FIGURE 5:**
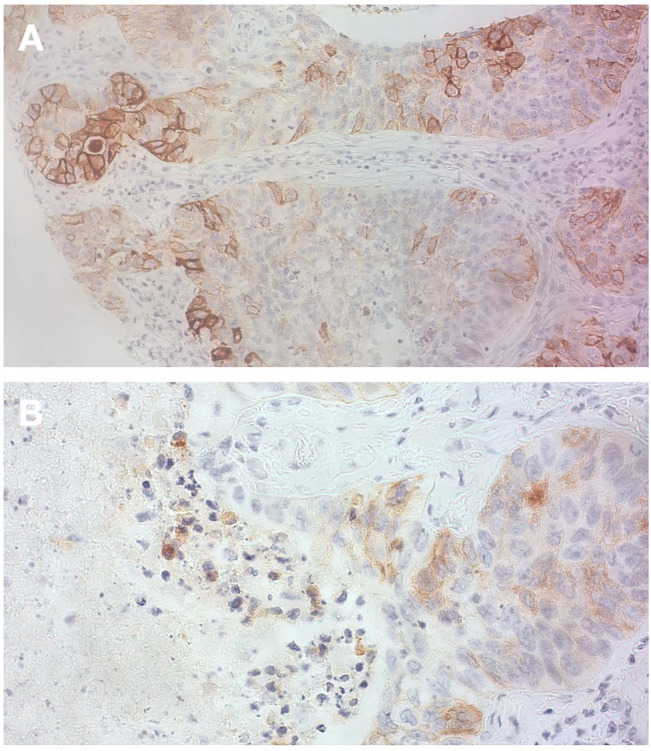
IHC analysis for PD-L1 **(A)** This picture highlights heterogeneity in term of PD-L1 expression in tumor cells (20 X magnification); **(B)** Weak PD-L1 positivity mainly on tumor cells (also macrophages and very few stromal lymphocytes are positive) (40 X magnification).

Figures 4, 5 show the expression of PD-L1 and vimentin in some tissue slides to highlight the differential expression of these biomarkers in tumor cells and infiltrate with immune cells.

## Discussion

In the last few years PD-L1 expression in tumor tissue increased its importance because of the availability of ICIs which target the PD-1/PD-L1 pathway. However, these drugs have not yet reached a role in resected non-metastatic NSCLC. Vimentin expression is not directly involved in the treatment of this neoplasm, but it has a key role in the EMT process, which seems to be involved in resistance to various cancer treatments. Its prognostic role has been widely studied in NSCLC patients. Few studies explored its association with PD-L1 expression (Asgarova et al., 2018).

We previously retrospectively analyzed data on patients with advanced NSCLC consecutively enrolled in a clinical study in our center. PD-L1 and vimentin expression were detected by immunohistochemistry (Bronte et al., 2021). We used ≥1% and ≥50% as cutoff values to define PD-L1 positivity on immune cells because these cutoff values are the same used on tumor cells in the clinical practice to select patients for immunotherapy. A weak positive association between PD-L1 and vimentin in advanced NSCLC suggests a potential interplay between these biomarkers. Moreover, given our previous results (Bronte et al., 2021), the established prognostic value of these biomarkers in other subset of disease, we investigated their expression in an earlier disease setting, non-metastatic NSCLC patients. We also explored a potential role of immune infiltrate in tumor tissue and analyzed the prognostic impact of these combined markers.

We found a weak positive correlation between PD-L1 and vimentin expressions in tumor cells as well as a correlation between the quantity of immune cells and % of immune cells PD-L1 positive. In particular, we retrospectively analyzed Vimentin and PD-L1 expression in tumor cells and immune infiltrate to study their separate and combined effects in terms of OS.

In our study a positive association between the percentage of PD-L1 positive tumor cells and the percentage of vimentin in tumor cells was seen.

The interplay between EMT markers and immune checkpoint inhibitors (Chouaib et al., 2014; Chen et al., 2015; De Matteis et al., 2019) has been previously reported. The concurrent expression of PD-L1 and EMT phenotype was described both in tumor tissue and circulating tumor cells (Kim et al., 2016; Raimondi et al., 2017; Manjunath et al., 2019). The effect of EMT on immune evasion is exerted through the regulation of PD-L1 expression (Asgarova et al., 2018) as a consequence of a synergistic exposure to TGF-β1 and TNF-α. These factors determine global DNA demethylation, also in the promoter of the gene encoding for PD-L1, and consequently a higher PD-L1 expression is achieved.

Ancel and colleagues reported that a cut-off ≥ 25% vimentin-positive tumor cells was significantly associated with poor tumor differentiation even if it was not sufficient to predict a worse prognosis. However, these authors reported that concurrent high PD-L1 and vimentin expressions in early-stage NSCLC patients were more clearly associated with a shorter OS (Ancel et al., 2019). Such results are concordant with our finding of a trend even if it was not statistically significant of a worse survival in the patients harboring double positivity for PD-L1 and Vimentin using 1% cutoff value for both the biomarkers.

Our study has some limitations, because it is retrospective and this population of non-metastatic NSCLC patients is heterogeneous, mainly in terms of disease stage, type of surgery and subsequent treatments. Another strong limitation is the use of archival tissue for IHC analysis and the limited number of patients included in this study. A prospective study could allow the analysis in fresh tissue to evaluate more biomarkers associated with EMT and immune evasion. Moreover, proper cut-off values to define vimentin and PD-L1 positivity have to be established. Probably, we did not find any prognostic significance of immune infiltrate because the overall amount of lymphocytes is not sufficient to determine a prognostic effect, but perhaps the specific subsets of lymphocytes (e.g. CD4^+^, CD8^+^, etc.) could impact on prognosis, but we could not determine these subsets because of the few slides available. Anyway, our findings suggest that EMT markers and immune escape markers could be intended as components of the same process.

## Data Availability

The raw data supporting the conclusion of this article will be made available by the authors, without undue reservation.

## Abbreviations

NSCLC, non-small cell lung cancer; IASLC, International Association for the Study of Lung Cancer; OS, overall survival; ICI, immune checkpoint inhibitor; PD-1, programmed death 1; PD-L1, programmed death ligand 1; IHC, immunohistochemistry; EMT, epithelial-to-mesenchymal transition; HR, hazard ratio; CI, confidence interval; KRAS, Ki-ras2 Kirsten rat sarcoma viral oncogene homolog; EGFR, epidermal growth factor receptor; ALK, anaplastic lymphoma kinase; ROS1, c-ros oncogene 1.
